# Hepatitis E Infection in a Renal Transplant Recipient

**DOI:** 10.1155/2014/865471

**Published:** 2014-09-11

**Authors:** Diana Vassallo, Mir Mubariz Husain, Shaun Greer, Stephen McGrath, Samreen Ijaz, Durga Kanigicherla

**Affiliations:** ^1^Department of Renal Medicine, Central Manchester NHS Foundation Trust, Manchester Royal Infirmary, Oxford Road, Manchester M13 9WL, UK; ^2^Department of Clinical Virology, Central Manchester NHS Foundation Trust, Manchester M13 9WL, UK; ^3^Department of Hepatology, Central Manchester NHS Foundation Trust, Manchester M13 9WL, UK; ^4^Department of Pathology, Central Manchester NHS Foundation Trust, Manchester M13 9WL, UK; ^5^Blood Borne Virus Unit, MS-Colindale, Public Health England, London NW9 5EQ, UK

## Abstract

An asymptomatic 35-year-old renal transplant recipient was noted to have deranged liver function tests. Liver biopsy revealed a portal inflammatory process with mild lobular activity and portal fibrous expansion, consistent with a virally mediated process. An extensive viral screen confirmed infection with Hepatitis E virus genotype 3 (HEV-3). There is increased awareness about locally acquired Hepatitis E virus (HEV) infection in the transplant population in the UK. The important implications of this infection are becoming more apparent as progression to liver cirrhosis can occur. However, the incidence, natural history, and treatment of HEV infection in the transplant population are not well established. This report illustrates a case of delayed spontaneous clearance of the HEV infection.

## 1. Introduction

In recent years, there has been increased interest in HEV infection as emerging data has revealed that chronic infection can lead to progressive liver disease in immunosuppressed patients including various transplant cohorts such as heart and lung transplant recipients [1, 2]. Chronic Hepatitis E should be considered in the differential diagnosis of elevated liver enzymes in transplant recipients. The frequency and course of these infections are however not well defined. The following case report highlights the challenges involved in diagnosis and treatment of this condition in a kidney transplant recipient and illustrates a case of delayed spontaneous clearance of the infection.

## 2. Case Report

A 35-year-old gentleman received a deceased donor renal transplant in 2006 following end-stage renal failure secondary to focal sclerosing glomerulosclerosis (FSGS). Basiliximab was used as induction immunosuppression followed by maintenance therapy with ciclosporin and prednisolone. Over the subsequent years, there was persistent moderate graft dysfunction with serum creatinine of 200 *μ*mol/L. Graft biopsies revealed chronic interstitial fibrosis and tubular atrophy. An episode of T-cell mediated rejection in 2009 (Banff 1A on biopsy) was treated with pulsed methylprednisolone and mycophenolate mofetil (MMF) was commenced. Graft function continued to deteriorate and in April 2010 ciclosporin was stopped. His immunosuppression at this stage consisted of MMF 1 g bid and prednisolone 5 mg daily.

**Figure 1:**
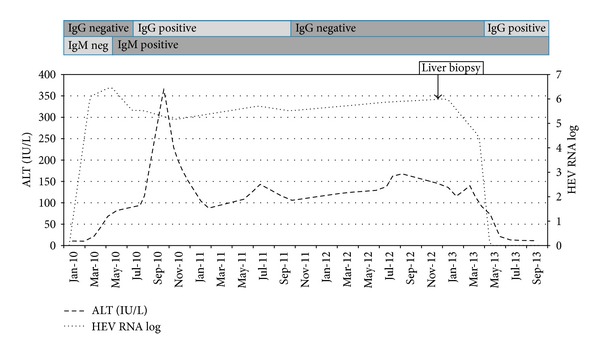
Evolution of alanine transaminase (ALT) levels, Hepatitis E virus (HEV) RNA levels, and HEV serology.

In April 2011 he was referred to the transplant clinic. As part of the work-up towards listing for subsequent kidney transplantation, deranged liver function tests (LFTs) were noted. A sharp rise in alanine transaminase (ALT) had occurred around April 2010 (Figure 1). There was no past history of jaundice, nor any change to his medication around this time. There had been no change in alcohol intake, nor any significant change in weight. He had gone on holiday to Tenerife in November 2009 and no illnesses had been reported during this trip. Clinical examination revealed a mildly obese gentleman with abdominal striae but there were no stigmata of chronic liver disease.

Ultrasound of the liver revealed a bright echotexture in keeping with fatty infiltration. Routine liver and immunology screen did not reveal any abnormality, including Hepatitis B, Hepatitis C, human immunodeficiency virus (HIV), cytomegalovirus (CMV), and Epstein-Barr viral (EBV) titres. The presumed diagnosis was steatohepatitis, although this did not explain the acute rise in LFTs. Further evaluation was not possible at this stage due to missed hepatology appointments, until a liver biopsy was performed in January 2013.

**Figure 2:**
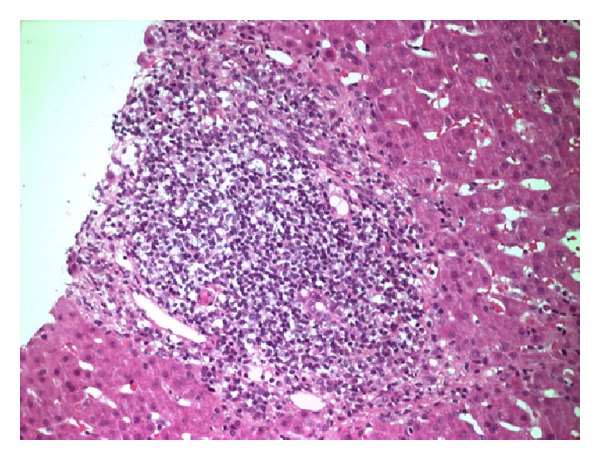
Liver biopsy showing a portal tract containing a dense, predominantly lymphocytic infiltrate. There is minimal inflammation affecting the peripheral interface region of the portal tract. The portal vein, hepatic artery, and several biliary structures can be seen within the infiltrate (haematoxylin and eosin stain, magnification ×20).

There was evidence of relatively mild fibrosis involving the portal tracts only, some of which appeared expanded and larger than usual. Features of more advanced fibrosis, such as bridging fibrosis linking adjacent portal structures and nodule formation, were not identified (Figure 2). The appearance was consistent with that of a virally mediated process. A more extensive viral screen was performed and serology was consistent with active Hepatitis E virus (HEV) infection. Detection of HEV antibody was carried out using the Wantai IgM and IgG detection assays (Fortress Diagnostics, Northern Ireland). The assays were run in accordance with the manufacturer's instructions. Subsequently, nucleic acid was extracted from serum using the Magna Pure 96 platform (Roche diagnostics Ltd, UK). HEV ribonucleic acid (RNA) detection and quantification were then undertaken via a real time assay [3]. This confirmed HEV genotype 3 (HEV-3) infection. HEV RNA detection and quantification were undertaken using qPCR but genotyping was done on a separate assay. This is based on PCR amplification and Sanger sequencing of a 300 base pair (bp) region across the open reading frame (ORF) 2 as published previously [4]. Retrospective serological and virological assays were performed on blood samples dating back to 2009.

The patient's immunosuppressive regime was not altered during this time. In April 2013 LFTs normalised spontaneously and the HEV RNA levels became negative (Figure 1). This remains negative to date. The patient started haemodialysis in January 2013 following graft failure and is now on the national waiting list for kidney transplantation.

## 3. Discussion

HEV infection in transplant recipients has gained more recognition in recent years. Emerging knowledge about this infection has raised concerns specific to this patient cohort [5]. In immunocompetent individuals, HEV generally manifests itself as a self-limiting acute viral hepatitis, with the exception of a fulminant presentation in pregnant women and in patients with chronic liver disease, although this has only been described in developing countries, mainly due to waterborne HEV genotype 1 [6]. However, it is known that HEV can lead to chronic hepatitis in approximately 60% of infected solid organ transplant recipients and 14.3% of these develop liver cirrhosis [5, 7, 8].

HEV is now recognised as an endemic or autochthonous infection in Europe and is no longer considered an imported infection from hyperendemic developing countries [9]. HEV-3 is distributed worldwide and is a porcine zoonosis associated with consumption of pork products [9, 10]. While on holiday in Tenerife, our patient consumed several pork-based meals. The incubation period in immunocompetent individuals is between 2 and 6 weeks [8] although this has not been characterised in transplant patients. It is not possible for us to distinguish between the Spanish and UK HEV-3 with confidence. However, given that in our report HEV RNA was detected more than 4 months following the patient's last holiday, it appears that likely the infection was acquired locally in the UK.

In the last few years, awareness of the HEV burden in developed countries, especially in the immunosuppressed cohort, has increased [11]. Most of the reported cases have been diagnosed in Southern France [12] where the incidence of HEV among liver, kidney, or simultaneous kidney-pancreas transplantation has been quoted as 3.2 cases/100 person-years [8]. In the UK, there is increased awareness of HEV infection amongst the general population and a public health issue was recognised in 2005 due to a significant number of infections in the general population [13]. A HEV-IgG seroprevalence rate of 16% has been detected in blood donors in Southwest England [14]. However, the incidence of HEV infection in transplant recipients in the UK is unknown.

Clinical suspicion is essential to enable a timely diagnosis. Transplant recipients are on a multitude of potentially hepatotoxic medication. In addition, HEV infections can be relatively asymptomatic with modest elevations in transaminases even in healthy individuals. Serological assays show a range of sensitivities and specificities and HEV serology may be negative in immunocompromised individuals [15]. Our patient became HEV-IgG positive around the time of detectable HEV RNA load; however, the IgG response was quite weak, just harbouring above the assay cut-off. HEV-IgG levels become undetectable in September, 2011, and detectable again in May 2013. However this is probably not reseroconversion but reflects fluctuation of a low IgG signal around the assay cut-off. The diagnosis of HEV can only be confirmed by performing HEV RNA detection studies [16]. HEV RNA testing is increasingly becoming more accessible and some commercial assays are now available. These diagnostic challenges raise the possibility that the burden of this infection in transplant recipients is underestimated.

Around 34% of immunosuppressed patients infected with HEV spontaneously clear the virus within 6 months, without any change in underlying immunosuppression or use of antiviral agents [7]. In our case, the patient cleared the virus spontaneously at approximately 36 months of infection. Delayed spontaneous clearance was reported in one other case in the literature, where the viral clearance occurred after 42.1 months [5]. It is not clear what triggered viral clearance in our patient as there was no change to his immunosuppression or in his clinical condition, at and a few months preceding the time of viral clearance, apart from the commencement of haemodialysis in January 2013.

In conclusion, HEV infection can be acquired locally and should be considered as a differential diagnosis of unexplained deranged liver function tests in transplant recipients. Screening for HEV by PCR should be routinely done in such cases. There is a need for more data regarding incidence and outcomes of HEV infection in transplant recipients in the UK. Factors that trigger spontaneous clearance of these infections are poorly understood. Increased awareness of locally acquired infections will help optimise management by enabling early diagnosis and contributing to knowledge about effective treatment and possible preventative measures.
